# Collectanea Medica, Consisting of Anecdotes, Facts, Extracts, Illustrations, Queries, Suggestions, &c.

**Published:** 1819-02

**Authors:** 


### COLLECTANEA MEDICA,

CONSISTING OF

### ANECDOTES, FACTS, EXTRACTS, ILLUSTRATIONS, QUERIES, SUGGESTIONS, &c.

RELATING TO THE

*History or the Art of Medicine, and the Auxiliary Sciences.*

Quicquid agunt medici, nostri farrago libelli.

SEVERAL of the medicinal and surgical measures employed by the natives of the *Tonga Islands* are so highly interesting, both from the nature of the diseases for which they were employed, and the marks of accurate and judicious observation they evince in the people of that singular nation, that we have no doubt our readers will be gratified as well as instructed by a relation of some of the most important of them.

We shall at present give an account of their mode of performing paracentesis of the thorax; or, as they term it, the *Cawso*:—*

Cawso is an operation which is performed to allow of the escape of extravasated blood, which has lodged in the cavity of the thorax in consequence of wounds, or for the extraction of a broken arrow. There are no other instances where they think of performing it. The instruments they use are a piece of bamboo and a splinter of shell; sometimes a probe made of the stem of the cocoa-nut leaf. Mr. Mariner has seen a number of persons on whom the operation had been performed, and who were in perfect health; and two instances of the fact itself he was an eye-witness to. The one we are about to describe was performed upon a Fiji islander, who had received a barbed arrow in the right side, between the fifth and sixth ribs; not in a line directly below the nipple, but about an inch backwards. The arrow had broken off about three inches from the point,† under the third row of barbs, and, from the rise and fall of the thorax in the act of respiration, the whole piece was perfectly concealed from any external view: the barbs and the point were of the same piece with the arrow.

---

* *From an Account of the Natives of the Tonga Islands, in the South Pacific Ocean;* by Mr. William Mariner. 2 vols. 8vo. Murray, London.

† They are made thin under each barb, on purpose that they may break. The barbs of this arrow were about a quarter of an inch transverse diameter, and the stem of the arrow under each row of barbs about the eighth of an inch.

A countryman of the wounded man wished to perform the operation; but the patient desired that a friend of his, a native of Vavaoo, should manage it: this proved that he placed at least equal confidence in his skill as in that of his countryman; for he had seen him perform the operation several times before, at the Fiji islands.

The patient was now lying on his back, but a little inclined to his left side; and this was considered a favourable posture for the operation. It was a fine clear day, and the weather warm: had it been rainy or cloudy, or had the patient felt himself cold, fires would have been lighted in the house, and a burning torch held to his side, to relax the integuments, and to render by such means the wound more favourable. The wound had been received the day before; and, on pressing the finger upon its orifice, the broken end of the arrow could not now be felt, except by the pain which such pressure gave the patient. In the first place, the operator marked with a piece of charcoal the situation and length of the intended incision, which was about two inches; the small wound made by the arrow being in the centre of it. The integuments were now drawn upwards, so that the black line lay upon and parallel with the superior rib; an assistant pressing his hand above, and another below, the situation of the intended incision, with a view to keep the integuments firm and steady. The operator having now chosen a fit piece of bamboo, began his incision, and carried it down to the bone, the whole length of the mark, which was done by five or six motions of the hand, aided by considerable pressure: in this part of the operation a shell could not be used, on account of its liability to break. The integuments being now allowed to return to their natural situation, the incision was cautiously continued with a splinter of shell, midway between the two ribs, dividing the intercostal muscles to nearly the same extent as the external wound, to allow of the introduction of a finger and thumb to lay hold of the arrow: during this part of the operation, however, the end of the arrow became perceptible, protruding between the costæ at every inspiration: the operator, as soon as possible, secured it with the finger and thumb of his left hand; whilst with his right he proceeded to widen the incision on either side, that he might take a deeper and firmer hold, and secure, if possible, the second row of barbs. To facilitate the operation, he now slipt the noose of a string over the barbs he held between his finger and thumb, and, having secured which, his left hand was no longer in the way of his right; for, by drawing the string as far as prudence would allow, he kept it prest upon the superior rib, and thereby preserved the arrow from receding at every expiration. The incision was now carried through the intercostal muscles and the pleura, sufficiently to allow of the introduction of the finger and thumb of the right hand, with which he endeavoured to disengage as much as possible what might obstruct the barbs; whilst with his left finger and thumb he laid hold of the end of the arrow, and kept gently twisting it, always one way, so as to break down those obstructions which could not be removed with the other hand, taking care, however, not to use so much force as might be supposed liable to break the barbs; and in this way, in the course of two or three minutes, he withdrew the arrow, bringing with it a small portion of the substance of the lungs, which could not be disengaged. During this part of the operation the patient was almost insensible: he was held by those about him, to prevent any mischief arising from his struggles, which at times were violent. The operator now carefully examined the arrow, and being satisfied that every barb (of which there were three rows) was entire, he ordered him to be gently turned on the right side, so that the wound was depending; and, to make it more completely so, a quantity of gnatoo was placed under him in two situations,—viz. under the shoulder and under the pelvis,—in such a way that the orifice of the wound was evidently the most depending portion of the thorax. The patient being now perfectly sensible, the operator desired him to make a full inspiration, enquiring whether it gave him much pain; and, being answered that he could bear it tolerably well, he desired him to make several full inspirations from time to time, but not so as to fatigue himself, and occasionally to move his body gently: by these means a considerable quantity of blood was discharged. A few hours afterwards, the operator introduced between the ribs a portion of banana leaf, smoothly folded several times, and anointed with cocoa-nut oil, as a pledget to keep open the wound. He ordered his patient to be kept perfectly quiet, not to be spoken to, no noise to be made, nor his attention to be attracted in any way: to live chiefly upon vegetable diet, or, if he had any kind of meat, fowl in preference to pork, or, if pork, it was to be very small in quantity, and without the least fat; with cocoa-nut milk for drink, in any quantity that he felt disposed to take. The first night he had a great deal of pain, much thirst, and little sleep; the following day he was much easier,—a great deal of blood was found to have been discharged, and a fresh pledget was introduced, which was renewed every morning as long as any discharge was apparent. When the discharge of sanguineous fluid ceased, which was in about nine or ten days, the operator introduced his probe, to be sure that the cessation of the discharge was not occasioned by any obstruction: he then contented himself with a more superficial pledget, that the external orifice might not heal too soon; and the patient was allowed to change his posture occasionally, but not for a long time together. As he grew better, a little more meat was allowed him; but the use of cava was interdicted until he got tolerably well. The wound healed in about six weeks, without any sort of dressing or washing. The patient was confined to his house about two months, and was not perfectly recovered till near a twelvemonth; when he seemed as healthy and as strong as ever, with scarcely any cough having supervened in the meanwhile. This was considered a very dangerous wound, and a very well-conducted cure. Mr. Mariner does not know that they are acquainted either with the exact situation or existence of the intercostal arteries.

It often happens that the arrow, not being a barbed one, is withdrawn without any difficulty; but still the surgeon thinks proper to perform the operation of *cawso*, not by enlarging the wound made by the arrow, but by making another at some little distance from it, in a part which, either from judgment or education, he deems more safe and proper. In all those persons whom Mr. Mariner knew to have undergone the cawso, it had been performed in nearly the same situation as the one above stated.

We have observed in the before-mentioned case that the wound was not washed; and it may here be noticed, that, in all cases of considerable wounds produced by pointed instruments, the patient is not allowed to wash himself till he is tolerably well recovered, nor to shave, cut his hair nor his nails: for all these things, they say, are liable to produce *gita* (tetanus), unless the wound be of such a nature, and in such a situation, that it may with safety be first laid completely open,—then there is no danger. Mr. Mariner never witnessed a case of tetanus produced by these means; but he met with many who said they had seen it in persons who had got nearly well of their wounds: but, happening to wash themselves too soon, spasm supervened, and death was the consequence. They notice that wounds in the extremities, particularly in the feet and hands, are liable to produce tetanus: also, in persons already wounded, sudden alarms, or even any sudden noise that calls the attention abruptly, is liable to produce this complaint. They never allow females to be near men thus wounded, lest the mere stimulus of venereal desire should induce this dangerous complaint. As to cutting the hair and nails, they positively assert that the mere sensation of these simple and common operations has not unfrequently been productive of these dreadful consequences. The man, whose case we have just mentioned, was eight months without being washed, shaved, or having had his hair or nails cut.

---

### *Case of Change of Colour of the Skin from Brown to White in a Native of Bengal.*

J. W. aged 56, a native of Bengal, his parents Mahometans, and both dark, left India about the age of ten or eleven, and has since resided in Edinburgh, chiefly performing the office of a servant; but since the last nine or ten years he has worked as a mason's labourer. During this period he has gradually lost his native dark colour, and become white, which he attributes partly to the climate, and partly to the action of lime and mortar in his occupation as a mason, which occasioned much itching of the skin. The change commenced in the hands and head; the hair, from being black and lank, has become light grey, and somewhat curled. The parts which last retained their colour were the breast and back of the neck. The only remains of his original complexion at present are some irregular patches of a dull purplish colour, covering the upper parts of the cheeks and prominences of the ears, and a lighter patch at the tip of the nose. During the change of his colour, no sensible alteration was observed in his health.*

*On Stimulants and Sedatives;* by Dr. WILSON PHILIP.†

IN compliance with your request, I trouble you with a few observations in illustration of the following remarks in the 254th page of the second edition of my Enquiry into the Laws of the Vital Functions; namely, "a moderate application of every agent appears to act as a stimulus; and excessive application of it as a sedative. The quantities which act as stimulus and sedative bear no particular proportion to each other, but in different agents exist in every possible proportion."

It appears, on the most cursory view of the phenomena of life, that they depend on a capacity of action in living parts, and the operation of agents capable of exciting them. Thus, the heart possesses the power of contraction, but it soon becomes inactive if the blood is withdrawn. The degree of excitement produced is proportioned to the force and continuance of the exciting cause and degree of excitability possessed by the part acted on. By the action of the stimulus, the excitability is always impaired, and by its continued action at length exhausted. Thus excitement continues, unless the agent is withdrawn, till the part is so far deprived of its excitability that it will no longer obey the same degree of the same agent. To produce further excitement, a more powerful agent must be applied, or the excitability of the part acted on must be increased.

The excitability is, within certain limits, increased by the abstraction of agents. Thus, for example, our sensibility (one species of excitability) is exhausted by the various agents which affect us during the day; and we find, by degrees, that the same agents no longer excite us. If more powerful agents are not applied, we soon fall into a state of insensibility—sleep, during which, the operation of the usual agents being withdrawn, we again become sensible to these agents.

Such are the more evident laws of excitability; and it would be easy, I think, to prove that they are the laws which regulate the cerebral system in a state of health. But it has been maintained that the same laws regulate the excitability of every part of the animal. To this position a very obvious objection occurs.

When the eye becomes wearied with seeing, the ear with hearing, &c. they cease to be excited; their excitability is thus allowed to accumulate, and they are again fitted for their functions: they

* *Edinburgh Clinical Reports*, p. 142.

† Communicated to Dr. Thomson, the editor of the Annals of Philosophy.

are not concerned in the preservation of life. An animal may be in perfect vigour, as far as relates to the powers on which his existence depends, although he neither sees nor hears. The vital powers remaining in sleep restore vigour to the exhausted organs of sense; but, were the vital organs themselves subject to similar exhaustion, what, during such intervals, would preserve the life of the animal? and by what powers would the vigour of these organs be restored?

It has been said, indeed, that the diastole of the heart arises from the stimulus of the blood exhausting its excitability in the systole which is restored to it during the interval that elapses between its contraction and the occurrence of that degree of distention which again excites it. But a very simple experiment shows the fallacy of this opinion.

If the heart is exhausted by the stimulus of the blood, and recovers its excitability during the absence of such a quantity of this fluid as is capable of exciting it, it ought not to recover its excitability if it is prevented from expelling any part of the blood which has excited it; for we have seen that the continued application of the same stimulus which has produced exhaustion cannot again excite the exhausted part, as no renewal of excitability can take place while the agent which exhausted it is still applied. The retina will never recover its powers under the impression of the same degree of light which exhausted it. We cannot recover from fatigue while the cause of our fatigue still operates. But the alternate contractions and relaxations of the heart still take place, as I have ascertained by repeated experiment, although a ligature be thrown round the aorta, in consequence of which the heart remains uniformly gorged with blood. The result of this experiment is not influenced by previously destroying the sensibility of the animal by a blow on the occiput.

If we sprinkle salt on a muscle, it does not occasion permanent contraction followed by exhaustion, but a constant alternation of contraction and relaxation, although the salt is never removed. The state of the muscle, however, in the relaxations which intervened between the contractions is evidently very different from that in which it is left when the salt can no longer excite any contraction in it.

The foregoing facts seem to prove that the nervous and muscular excitability obey different laws. While the effect of uniform stimuli acting on the former is permanent excitement, followed by exhaustion, the habit of the muscular fibre under the influence of uniform stimuli is to act by intervals. This is probably the cause why moderate excitement seems not to exhaust the muscular fibre, while the nervous fibre suffers proportional exhaustion from every degree of excitement.

Two circumstances appear to be capable of occasionally counteracting this habit, and producing in the muscular fibre permanent contraction, a peculiarly strong stimulus, and the influence of the will. It is only, however, occasionally that the most powerful stimuli have this effect; and it is only for a limited time that the will can produce it. After a certain time, the natural tendency of the muscle to alternate contraction and relaxation prevails, and the limb which we wish to keep steady begins to tremble.

There is another species of debility of the living fibre, of a very different nature from the exhaustion we have been considering, which appears to bear no relation to any previous excitement, but to be the direct effect of agents; for, while some agents increase, others lessen, the action of this solid. The former have been called stimulants, the latter sedatives.

It has been maintained, indeed, that, as exhaustion is the effect of moderate excitement, the species of debility we are now considering is always the consequence of excessive excitement; and, therefore, that, like exhaustion, the sedative effect is never the direct effect of the agent. And this opinion seems, at first view, to be countenanced by the fact that stimuli act as sedatives when applied in excess. Thus, a moderate quantity of distilled spirits received into the stomach produces excitement, which, within certain limits, is greater in proportion to the quantity taken; but, if a very large quantity be suddenly received into the stomach, it produces no degree of excitement, but immediate debility, or even death.

It is surely a strained explanation of the latter effects, however, to suppose them the consequence of excessive excitement,—no symptom of which appears. The supposed existence of this excitement rests wholly on the preconceived opinion that exhaustion, in consequence of previous excitement, is the only debility which can arise from the operation of agents on the living fibre. It has, therefore, been maintained that, however imperceptible the excitement produced by large quantities of distilled spirits, for example, we must suppose that their first effect is excitement, and their debilitating effect, consequently, only secondary. And so much has this idea laid hold of the minds of many, that, in an account of the above Enquiry, lately published in a journal of great respectability, my opinion of the nature of inflammation is opposed on the ground that the operation of agents in producing this disease must, in the first instance, be stimulant; and the debility of the vessels, which it is admitted exists in inflamed parts, the consequence of previous excitement; and this is maintained without questioning the authority of my experiments, from which it appears that none of this previous excitement could be perceived with the aid of powerful microscopes. Now, I may surely be allowed to maintain that, where no increased action can be perceived, none should be supposed. We must not substitute hypothesis for plain matter of fact.

But, if this argument, which is of all the most conclusive, were out of the question, there is another, which (as far as I can judge) would be unanswerable, to which even the supporters of the hypothesis in question must listen. It must be admitted, even by them, that, if the tendency of different agents to produce debility arises from the degree of excitement occasioned by their first impression on the living solid, those best calculated to produce excitement should be found capable of the greatest sedative effect. But this is so far from being true, that we find that the agents which produce the greatest degree of this effect are the worst stimuli. Tobacco, for example, which is one of the most powerful sedatives, cannot, by any management, be made to produce the degree of excitement which arises from opium or distilled spirits.

The sedative, like the stimulant effect, may be communicated to the muscular through the nervous system. When tobacco is applied to any considerable part either of the brain or spinal marrow, as I have ascertained by repeated experiment, the heart soon begins to act more languidly; but this languor is preceded by little, if any, increased action, unless the tobacco be applied in a very diluted state; in which case, it produces comparatively little languor, and the excitement is much less than that produced by opium, which is followed by no sensible languor.

If we disregard pre-conceived opinions, and fix our attention on facts alone, we shall, as far as I am capable of judging, arrive at the following conclusions.—Every agent capable of affecting the living solid acts both as a stimulus and sedative, according to the degree in which it is applied. Applied within certain limits, it is a stimulus; and, in proportion as it stimulates, it exhausts the excitability,—this being equally true of the interrupted excitement which stimuli produce in the muscular, as of the permanent excitement which they produce in the nervous, system. Applied beyond these limits, agents no longer produce excitement followed by proportional exhaustion, but direct exhaustion arising from the operation of the agent, and wholly unconnected with previous excitement,—the stimulant and sedative powers existing in no particular proportion to each other, but in different agents in every possible proportion. I have just had occasion to mention the comparative effects of tobacco and opium in the heart. Thus, the stimulant power of distilled spirits is great, its sedative power small. It must be used in very great quantity to produce the sedative effect; while a small quantity of digitalis produces this effect, and its stimulant power is very slight.

These observations apply to agents affecting the mind as well as the body. Grief and fear possess great sedative power; they act as stimulants only when they are present in a comparatively small degree. Joy and anger, on the other hand, are powerful stimuli, and only act as sedatives when in excess. There is no exception, I believe, to the law we are considering. There is no agent which may not be made to produce a stimulant effect by applying it in very small quantity, and none which does not act as a sedative when applied in excess.

